# A multidisciplinary approach for people with HIV failing antiretroviral therapy in South Africa

**DOI:** 10.4102/sajhivmed.v25i1.1579

**Published:** 2024-07-22

**Authors:** Parisha M. Juta, Juan M. Jansen van Vuuren, Kabamba J. Mbaya

**Affiliations:** 1 Department of Internal Medicine, Faculty of Health Sciences, School of Medicine, University of KwaZulu-Natal, Pietermaritzburg, South Africa; 2 Department of Internal Medicine, Faculty of Health Sciences, School of Medicine, University of KwaZulu-Natal, Durban, South Africa; 3 Department of Internal Medicine, Joint Royal Colleges of Physicians Training Board, National Health Service (NHS) England, Chelmsford, United Kingdom; 4 KwaZulu-Natal Department of Health, Northdale Hospital, Pietermaritzburg, South Africa; 5 Department of Family Medicine, School of Nursing and Public Health, University of KwaZulu-Natal, Durban, South Africa

**Keywords:** HIV, multidisciplinary, antiretroviral therapy, highly active antiretroviral therapy (HAART), multidisciplinary team, failure, patient centred

## Abstract

**Background:**

South Africa (SA) has the largest antiretroviral therapy (ART) programme worldwide. Multiple factors contribute to virological failure (VF), including poor adherence and viral resistance mutations. A multidisciplinary team (MDT) clinic dedicated to those with VF may be of benefit; however, very little data from SA exist.

**Objectives:**

To assess whether an MDT approach achieved virological suppression (VS) in patients failing second-line-ART (2LART); assess the number of MDT sessions required to achieve VS; assess local resistance mutation patterns and whether the MDT reduced the number of genotypic resistance testing (GRT) required.

**Method:**

An observational, retrospective, cross-sectional chart review study was conducted between January 2018 and December 2019 at a Target High Viral Load (VL) MDT clinic in KwaZulu-Natal, SA.

**Results:**

Ninety-seven medical records were eligible. Women accounted for 63% of patients, with a mean age of 37 years. A significant reduction in the first VL measurement following the MDT was seen (median reduction 2374 c/mL; *P* < 0.001). This was maintained at the second VL measurement post-MDT (median reduction 2957 c/mL; *P* < 0.001). Patients attended a mean of 2.71 MDT sessions and 73.2% achieved VS, resulting in 61.86% fewer GRTs required. Of the GRTs performed, nucleoside reverse transcriptase inhibitors and non-nucleoside reverse transcriptase inhibitor-related mutations were noted most frequently.

**Conclusion:**

The MDT approach resulted in a significant reduction in VL, with most participants achieving VS. The MDT was successful in reducing the need for GRT. Resistance mutations were similar to those found in other studies conducted across SA.

**What this study adds:** The findings of this study should serve to inform other clinicians, especially those in similar resource-limited settings, of the benefits of utilising a multidisciplinary approach to provide holistic, biopsychosocial care for people failing second-line ART to improve clinical outcomes.

## Introduction

The number of people living with HIV (PLHIV) was estimated to be 39 million in 2022, when 76% were accessing antiretroviral therapy (ART).^1,2^ The global community has significantly progressed in reducing the new infection rate (52% decrease since the peak in 1997), improving access to treatment and reducing AIDS-related deaths (64% reduction since 2004).^3^ The latest Joint United Nations Programme on HIV and AIDS (UNAIDS) targets state that by 2025, 95% of PLHIV will know their status, 95% of those diagnosed will be receiving ART, and 95% of people receiving ART will achieve virological suppression (VS).^2^

HIV and AIDS is one of the top five leading causes of death in Africa and South Africa (SA).^4,5^ It is estimated that SA has a population of 7.8m PLHIV, with an adult prevalence of 20%.^6^ This makes SA home to the largest ART programme in the world, with 5.6m people on treatment, of which only 66% are virologically suppressed.^3^ The setting of this study was Northdale Hospital (NDH), a district hospital in Pietermaritzburg, KwaZulu-Natal (KZN). This district hospital on the east coast of SA has the highest burden of HIV infection in the country, with a 24% prevalence and 1.2m people on ART.^7^ SA has implemented the 95-95-95 strategy and adopted the United Nations resolution to end the AIDS epidemic by 2030. Therefore, it is critical to achieve high levels of population VS.^8^

Viral load (VL) is a direct marker of active viral replication with inference on disease activity and progression.^9^ One of the aims of ART is to achieve VS (defined as less than 50 copies/mL [c/mL]).^10^ VS prevents disease progression, reduces transmission and minimises the acquisition of resistance to ART, all of which contribute to a reduction in AIDS-related morbidity and mortality.^10,11^ The South African National Department of Health (NDoH) defines virological failure (VF) on a protease inhibitor (PI)-based regimen, usually as second-line ART (2LART), as a VL > 1000 c/mL on at least three occasions over the course of 2 years, or a VL > 1000 c/mL with signs of immunological and clinical failure.^10^ With the longer duration of ART exposure in PLHIV, first- and second-line treatment failure is expected to increase due to the emergence of drug resistance and suboptimal ART adherence.^12^ Second-line failure is associated with a high mortality rate.^13^

In 2017, The South African National HIV Prevalence, Incidence, Behaviour and Communication Survey (SABSSM) showed approximately 84.8% of PLHIV within the 15-year-old to 64-year-old age group had tested for HIV and knew their status; 70.7% of this group were on ART and 87.4% of the group on ART achieved VS.^14^ At the time of the survey, SA was on track with meeting the first 90% target of the UNAIDS 90-90-90 targets for 2020, but fell behind on the second 90% target by approximately 20%. Although VS amongst those who were on ART was close to the third 90% target, the deficiency in those who were initiated and maintained on treatment cannot be ignored.

HIV can rapidly regenerate new variants due to its high mutation rate, which allows the virus to avidly replicate in the presence of ART.^15^ This promotes evasion of the immune system and the development of antiretroviral drug resistance-acquired mutations (DRAMs), which hinders the ability to achieve virological and epidemic control. ART efficacy is dependent on the genetic barrier to resistance which is defined as the number of HIV mutations required to develop resistance to an individual ART drug in the regimen. Poor adherence has been directly linked to a higher prevalence of drug resistance.^16^ Various studies have shown that high-income countries using more modern ART with a medium-to-high genetic barrier to resistance have a declining prevalence and incidence of drug resistance, whereas major ART roll-out programmes in LMICs, utilising regimens with lower genetic barriers, are promoting the development of resistance.^17,18^ The World Health Organization recommends an HIV drug resistance surveillance strategy for resource-constrained settings, including a combination of monitoring early warning indicators of poor programme performance, surveillance of transmitted drug resistance and evaluation of acquired HIV-1 drug resistance.^19^ Dolutegravir (DTG) has been included in the first-line ART regimen in SA as of 2020. Due to its high genetic barrier to resistance, the latest SA NDoH guidelines of 2023 recommend a lower adherence rate of more than 80% to achieve optimal clinical outcomes.^20^

Genotypic resistance testing (GRT) in HIV is used to detect known DRAMs in the enzymatic targets of ART.^21^ The European Guidelines formulated by the European HIV Drug Resistance Guidelines Panel, the US Department of Health and Human Services (DHHS) and the International AIDS Society (IAS) recommend drug-resistance testing at diagnosis before the initiation of ART.^22,23,24^ These guidelines are supported by numerous studies such as the VIRADAPT randomised control trial (RCT), VIRA3001 RCT and the Havana study, all of which emphasise the clinical efficacy of resistance testing to guide initial therapy and to optimise ART in those with VF.^25,26,27^ The SA NDoH guidelines do not recommend GRT in newly diagnosed PLHIV before the commencement of ART unless they have received pre-exposure prophylaxis (PrEP) in the last 6 months.^20^ GRT has been shown to be cost-effective in the United States and Europe; however, due to resource constraints, LMICs are unable to meet the same standard.^28,29^ The expansion of GRT in a resource-limited setting is hampered by high capital costs, quality assurance requirements, limited infrastructure, and a shortage of highly skilled and experienced personnel.^30^

At the time of the study, in the pre-DTG era, the SA NDoH Guidelines stated that any patient failing a PI-based regimen with confirmed VF is eligible for GRT.^10^ PI-based regimens have a high genetic barrier; therefore, resistance develops more slowly and an elevated VL on this regimen is more likely due to poor adherence.^10,15^ For this reason, PLHIV should be on a PI-based regimen for at least 2 years before undergoing GRT. It also allows for GRT in PLHIV with an elevated VL on a PI-based or DTG-based regimen (regardless of the time on regimen), who received concurrent Rifampicin-containing tuberculosis treatment and unboosted ART. A boosted ART regimen contains a drug that acts as a pharmacokinetic enhancer to increase the effectiveness of the primary drug in the regimen.^31^

A multidisciplinary team (MDT) approach in healthcare can be described as the mechanism for organising and coordinating health and care services to meet the needs of individuals with complex care needs.^32^ Studies have found better quality of care, improved clinical outcomes, patient safety, continuity of care and a more holistic approach towards individual needs using an MDT approach.^33,34,35^ The team can comprise a variety of professionals from different disciplines. This brings together diverse skill sets and expertise to assess, plan and manage an individual using the biopsychosocial (BPS) model approach.

Multiple studies which assessed an MDT approach in relation to ART treatment outcomes (specifically VS) have been conducted globally and have shown positive results.^36,37,38^ Elgalib et al. conducted a study in Muscat, which evaluated an MDT approach to outcomes in PLHIV.^36^ The MDT consisted of counsellors, nurses, social workers, pharmacists and doctors, and the study compared VS rates between December 2015 and June 2017. Overall, rates of VL < 200 c/mL increased from 71.9% in 2015 to 90.5% in 2017. Nijhawan et al. assessed the outcomes of inpatients receiving an MDT approach to their care in the United States.^37^ They found a 30% improvement in both the VS rates and engagement in care post discharge when compared to those who did not receive the MDT interventions. Onoya et al. identified patients at high risk for failing 2LART in Johannesburg, SA.^38^ VF was predicted by complex socioeconomic factors that contributed to poor adherence. Fogarty et al. summarised over 200 variables regarding patient adherence to ART into four broad categories, namely: factors related to the ART regimen, psychosocial factors, institutional resources, and personal attributes.^39^ This further emphasises that adherence to ART is complex and multi-level and requires a similar strategy to address its challenges.^36,37,38,39^

A study done in Johannesburg, SA, by Fox et al. looked specifically at patients failing 2LART and the role of intensive adherence counselling.^40^ This was a single-arm study of PLHIV on second-line PI-based ART with VL > 400 c/mL. Four hundred patients underwent intensive, targeted adherence counselling after having an elevated VL on 2LART; 97% then underwent repeat VL testing and 64% resuppressed (VL < 400 c/mL) on 2LART. Of those that remained unsuppressed, 41 out of 48 (85%) were found to have resistance on GRT.

In resource-limited settings such as the setting of this study, where GRT is expensive and poor adherence is the most common cause of treatment failure, it is crucial to develop a systematic approach to handling PLHIV with treatment failure, specifically to 2LART.^15,36,37,38,39,40^ Although third-line ART drugs are available in SA’s public sector, they come with a significant cost increase compared to first-line and 2LART regimens.^41^ Adequately identifying risk factors for failure on 2LART remains of utmost importance to reduce the need for third-line drugs. Whilst there is sufficient evidence emphasising the importance of intensive adherence counselling and various strategies to promote adequate adherence to ART exist, there is a paucity of data, especially in SA, on the impact and effectiveness of the currently employed strategies and specifically on the role of a MDT approach, whereby individualised, holistic patient-centred care can be provided to PLHIV who are facing difficulties with different aspects of the multi-level factors that contribute to non-adherence, achieving VS, preventing progression of the disease and the development of resistance.

In 2018, the district hospital’s specialised HIV, AIDS, tuberculosis (TB) and sexually transmitted infection (STI) clinic implemented a target high-VL clinic, now referred to as the Target Clinic, which utilises an MDT approach to address PLHIV with VF on 2LART. This clinic functions as a form of differentiated care for PLHIV with high VL and focuses on enhanced case management using a patient-centred approach. Patients are also empowered to make joint decisions with their healthcare providers regarding their treatment to improve ART adherence. Patients undergoing GRT are selected from this specialised clinic, if they are still considered to be failing 2LART after adequate MDT counselling sessions and good adherence is noted. VS was defined as a VL < 50 c/mL. VF on a PI-based regimen (usually present in 2LART) was defined as a VL > 1000 c/mL on at least three occasions over the course of 2 years or a VL > 1000 c/mL with signs of immunological and clinical failure.^10^ The definition of PLHIV who are defaulters or loss to follow-up (LTFU) differs between settings and has evolved over time.^42^ In the context of this, a defaulter was defined as any client on ART who missed a scheduled appointment or did not have ART drugs in hand for more than 90 days and this was in keeping with the guidelines at the time.^10^ Since then, the definition of a ‘defaulter’ has evolved to allow for increased accuracy of reporting data.^43^ Notably, in the latest South African NDoH’s National Consolidated Guidelines of 2023, the term ‘unconfirmed LTFU’ is used to describe a client who has not had ART drugs in hand for more than 90 days.^20,43^ However, for the purposes of this study, the term ‘defaulter’ was used.

### Objectives

#### Primary objective

To determine the effects on virological outcomes of a multidisciplinary, targeted clinical approach in PLHIV identified as failing 2LART at a district healthcare facility in Pietermaritzburg, KZN.

#### Secondary objectives

- To determine, in patients who achieve VS without undergoing GRT or changing antiretroviral regimen, the average number of MDT sessions needed to achieve VS.
- To assess whether a multidisciplinary, targeted clinical approach in PLHIV, identified as failing 2LART, improves adherence and reduces the need for expensive genetic resistance tests.
- To evaluate results of patients who had already undergone GRT. The specific gene mutations found were recorded and compared to the available literature on mutation patterns in SA for descriptive purposes.

## Research methods and design

An observational, retrospective, chart review cohort study was conducted at the Thembalethu Wellness Clinic, a specialised HIV, AIDS, TB and STI clinic located at a district hospital in Pietermaritzburg, KZN. The Target Clinic occurred weekly and comprised advanced MDT sessions which integrated care between a Family Medicine Consultant, a junior doctor or medical officer, a social worker, and an adherence counsellor. The Target Clinic had existing inclusion and exclusion criteria in place.

Inclusion criteria:

- HIV positive on 2LART currently
- VL > 1000 c/mL on at least two consecutive occasions taken at least 3 months apart
- minimum of 2 years on a PI-based ART regimen
- 14 years and older.

Exclusion criteria:

- HIV positive on first-line ART
- VL > 50 but < 1000 c/mL on any occasion
- VL > 1000 c/mL on only one occasion
- under 14 years old.

Demographic and clinical data were obtained from medical records of all PLHIV who attended the Target Clinic from January 2018 to December 2019. Demographic data including age, gender (assigned at birth), employment status, and living area were extracted. Clinical data included time since diagnosis, duration on ART, previous and current ART regimens, history of defaulting with motives (where applicable), history of opportunistic infections including tuberculosis and Hepatitis B, and concomitant comorbidities. Neuropsychiatric conditions were included under concomitant comorbidities and encompassed a broad range of neurological and psychiatric conditions. HIV-associated neurocognitive disorder (HAND), HIV-associated dementia, and mental healthcare users are included in this term. However, due to the perceived stigma associated with mental healthcare users, especially in the South African setting, the term ‘neuropsychiatric’ was preferred.^44^ Laboratory data were obtained from medical records and the electronic National Health Laboratory Service (NHLS) database.

The following variables were collected:

- The last CD4+ T-lymphocyte count (cells/mm^3^) in the period prior to commencement of the MDT sessions (pre-MDT) and the first CD4 count following MDT input (post-MDT).
- HIV VL (c/mL) was recorded at multiple, scheduled timepoints. Three pre-MDT and at least two following the initiation of the MDT approach (post-MDT).
- GRT results.
- TB GeneXpert (GXP) results.
- Hepatitis B surface antigen (HBsAg) results.
- Cryptococcal antigen results (where indicated).

Insight into local resistance patterns was gained by analysing the results of patients who have formed part of the Target Clinic and who have already undergone GRT. The specific gene mutations found were recorded and reported on to describe the profile of DRAMs found in our setting and to compare it with the profile of DRAMs described amongst patients failing 2LART in similar settings, from the local literature in SA. Notably, no additional GRTs were performed and therefore no additional costs were incurred.

### Statistical analysis

Descriptive statistics were used to evaluate demographic and clinical characteristics of the study cohort. The frequency distributions of all variables were examined by a registered statistician from the University of KwaZulu-Natal. Means with standard deviations (s.d.) were used to report normally distributed data.

VL data for varied timepoints showed evidence of skewing; therefore, they were summarised using the median and interquartile range (IQR) and were further divided into three categories:

- VL < 50 c/mL – PLHIV who achieved VS.
- VL 50–1000 c/mL – PLHIV who had a low-grade viraemia.
- VL > 1000 c/mL – PLHIV who still had VF.

Paired analyses were performed primarily utilising nonparametric tests. Due to the sample size, Fisher’s exact test was used to identify associations. The cohort was also grouped based on the results from the GRT. Further pairwise comparisons were performed where necessary. The Wilcoxon matched-pairs Signed-Rank and the Mann Whitney U tests were utilised to compare variables.

A *P*-value of less than 0.05 was regarded as statistically significant at a 95% confidence interval. Analyses were performed using Stata version 17 statistical software with the assistance of a statistician from the University of KwaZulu-Natal’s College of Health Sciences’ Biostatistics Department.

### Ethical considerations

Ethical clearance to conduct this study was approved by Northdale Hospital management, the provincial gate-keeping authorities and the Biomedical Research Ethics Committee, University of KwaZulu-Natal, School of Medicine (reference number: BREC/00004504/2022), prior to commencement of the study. Confidentiality and continued respect were of utmost importance throughout the study to protect the patients’ data and avoid further HIV-related stigma. Identifiers were in place and only the researchers had access to the raw data, thus ensuring that personal information was kept anonymous and safe guarded. These data will be safeguarded by the author in a password- protected electronic file for a period of 5 years, after which they will be discarded. There was no physical interaction with any of the patients and data were kept anonymous thereby negating the need for informed consent.

## Results

### Study population

**FIGURE 1:**
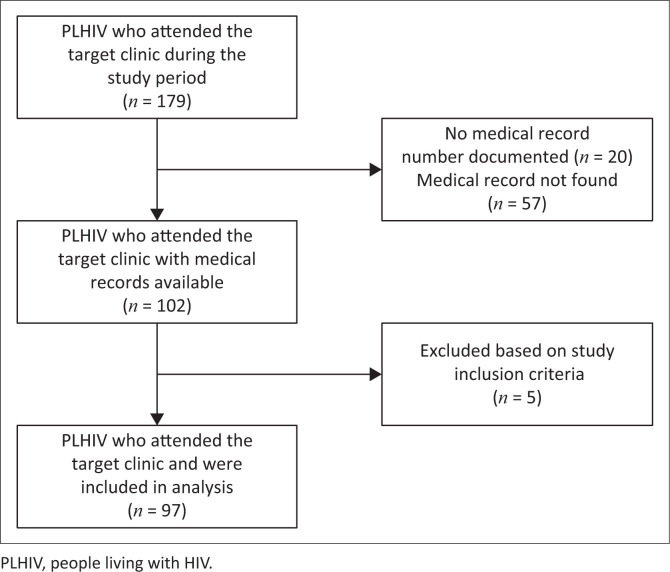
Preferred Reporting Items for Systematic Reviews (PRISMA) flowchart indicating final study cohort of PLHIV failing second-line ART.

A total of 179 PLHIV attended the Target Clinic over the 2-year study period. Following appropriate exclusions, 97 formed part of the study cohort (Figure 1). Notably, 5 PLHIV who were on first-line ART were exceptionally selected by their treating physician to attend the Target Clinic during the study period due to the numerous psychosocial challenges they faced at the time. These patients may have still benefited from the collaborative care provided by the MDT; however, they did not meet the study inclusion criteria of being on 2LART and were therefore excluded.

### Demographics and characteristics

Table 1 shows the demographic and clinical characteristics of the study population. Women accounted for nearly two-thirds of the cohort. The mean time since diagnosis was 14 years, and mean duration on ART was 13 years. Participants were on a second-line regimen for a mean of 7 years, with the most common regimen being a combination of Zidovudine (AZT), Lamivudine (3TC) and Lopinavir/ritonavir (LPV/r).

**TABLE 1:** Baseline characteristics of people living with HIV who attended the Target Clinic in 2018–2019, comparing male and female participants.

Baseline characteristics	*N*				Male participants				Female participants			
	*n*	%	Mean	s.d.	*n*	%	Mean	s.d.	*n*	%	Mean	s.d.
***N***	97	100.00	-	-	36	37.11	-	-	61	62.88	-	-
**Age (years)**	-	-	37.34	12.85	-	-	35.52	13.87	-		38.26	11.88
**Living area**												
Rural	42	43.29	-	-	18	50.00	-	-	24	39.34	-	-
Urban	51	52.58	-	-	17	47.22	-	-	34	55.73	-	-
Unknown	4	4.12	-	-	1	2.78	-	-	3	4.91	-	-
**Employment status**												
Employed	6	6.19	-	-	4	1.11	-		2	3.28	-	-
Self-employed	52	53.61	-	-	17	47.22	-	-	35	57.38	-	-
Unemployed	26	26.81	-	-	10	27.78	-	-	16	26.22	-	-
Unknown	13	13.40	-	-	8	22.22	-	-	5	8.20	-	-
Years since diagnosis	-	-	13.86	4.71	-	-	13.00	5.46	-	-	14.22	4.41
Years on ART	-	-	13.18	3.92	-	-	12.81	3.96	-	-	13.40	3.92
Years on 2LART	-	-	7.42	2.07	-	-	7.04	2.18	-	-	7.66	1.96
**Types of 2LART regimens**												
AZT + 3TC + LPV/r	54	55.67	-	-	23	23.71	-	-	31	31.96	-	-
AZT + 3TC + ATV/r	16	16.50	-	-	5	5.15	-	-	11	11.34	-	-
ABC + 3TC + LPV/r	7	7.22	-	-	2	2.06	-	-	5	5.15	-	-
TDF + 3TC + LPV/r	5	5.15	-	-	0	0.00	-	-	5	5.15	-	-
TDF + 3TC + ATV/r	4	4.12	-	-	1	1.03	-	-	3	3.09	-	-
AZT + DDI + LPV/r	2	2.06	-	-	1	1.03	-	-	1	1.03	-	-
AZT + 3TC + LPV/r + TDF	3	3.09	-	-	2	2.06	-	-	1	1.03	-	-
ABC + 3TC + ATV/r	1	1.03	-	-	0	0.00	-	-	1	1.03	-	-
AZT + 3TC + DTG	1	1.03	-	-	1	1.03	-	-	0	0.00	-	-
TDF + FTC + LPV/r	1	1.03	-	-	0	0.00	-	-	1	1.03	-	-
TDF + 3TC + DTG	1	1.03	-	-	1	1.03	-	-	0	0.00	-	-
TDF + EFV + ATV/r	1	1.03	-	-	0	0.00	-	-	1	1.03	-	-
DTG + 3TC + LPV/r	1	1.03	-	-	0	0.00	-	-	1	1.03	-	-

ABC, Abacavir; ATV/r, Atazanavir/ritonavir; AZT, Zidovudine; D4T, Stavudine; DDI, Didanosine; EFV, Efavirenz; FTC, Emtricitabine; LPV/r, Lopinavir/ritonavir; TDF, Tenofovir; 3TC, Lamivudine.

The latest pre-MDT results showed a median VL of 20 700 c/mL (IQR 4480–94 950), with a mean CD4 of 377.96 (s.d. 340.3) (Table 2). According to the guidelines at the time, 45 (46.39%) participants met the criteria for VF.

**TABLE 2:** Virological characteristics of the study cohort pre and post MDT intervention.

Virological characteristics	*N*						Male participants						Female participants					
	*n*	%	Median	IQR	Mean	s.d.	*n*	%	Median	IQR	Mean	s.d.	*n*	%	Median	IQR	Mean	s.d.
**Pre MDT**																		
Pre-MDT VL #1	85	-	9180	1820–37 800	-	-	31	-	13 000	2000.0–57 600.0	-	-	54	-	7654	1360–36 500	-	-
Pre-MDT VL #2	97	-	8820	1680–86 800	-	-	33	-	8970	1480.0–51 945.0	-	-	57	-	7350	1300–86 200	-	-
Pre-MDT VL #3	97	-	20 700	4480–94 950	-	-	36	-	14 800	2860.0–95 900.0	-	-	61	-	31 300	6640–94 050	-	-
VF Pre-MDT	45	46.40	-	-	-	-	10	27.78	-	-	-	-	35	57.38	-	-	-	-
Pre-MDT CD4	81	-	-	-	377.96	340.30	27	-	-	-	315.33	204.60	54	-	-	-	409.27	388.93
**Post MDT**																		
Post-MDT VL #1	97	-	3880	350–21 400	-	-	36	-	11 660	869.5–33 200.0	-	-	61	-	3880	241–17 850	-	-
Post-MDT VL #2	94	-	1225	50–16 000	-	-	36	-	1490	50.0–24 611.5	-	-	54	-	1315	50–16 300	-	-
Post-MDT CD4	38	-	-	-	331.18	321	16	-	-	-	270.50	347.50	22	-	-	-	344.77	285.63
Number of MDTs attended	-	-	-	-	2.71	3.63	-	-	-	-	3.94	5.48	-	-	-	-	1.98	1.43
Achieved VS post MDT	71	73.20	-	-	-	-	27	38.03	-	-	-	-	44	61.80	-	-	-	-
MDTs attended by those who achieved VS	-	-	-	-	2.71	3.39	-	-	-	-	3.74	5.00	-	-	-	-	2.09	1.58

MDT, multidisciplinary team; VL, viral load; VF, virological failure; VS, virological suppression.

Male participants had a higher median VL across the study period as well as a lower CD4 count when compared to female participants (Table 2). At least one additional comorbidity was found in 45 PLHIV (46.39%), with neuropsychiatric disorders being most common, and hypertension the second-most common.

There was a statistically significant reduction in the first VL measurement following the MDT intervention, with a median reduction of 2374 c/mL (IQR = 1180–31 840; *P* < 0.001). This was subsequently maintained at the second VL measurement post-MDT, with a median reduction of 2957 c/mL (IQR = 430–59 800; *P* < 0.001). A total of 71 PLHIV (73.20%) achieved VS and attended a mean of 2.71 (s.d. 3.39) MDT sessions (Table 2). Additionally, a reduction in mean CD4 count was noted post-MDT.

Individuals on a current regimen containing ABC, 3TC and LPV/r demonstrated a statistically significantly higher rate of achieving VS at 71.40%, when compared to those on regimens containing AZT, 3TC and ATV/r or LPV/r, at 25.00% and 26.90%, respectively (*P* = 0.01). This suggests a significant association between the current regimen of ABC, 3TC and LPV/r and achieving VS. However, these results should be interpreted within the context of the small sample size, where only five individuals achieved VS on this regimen.

**FIGURE 2:**
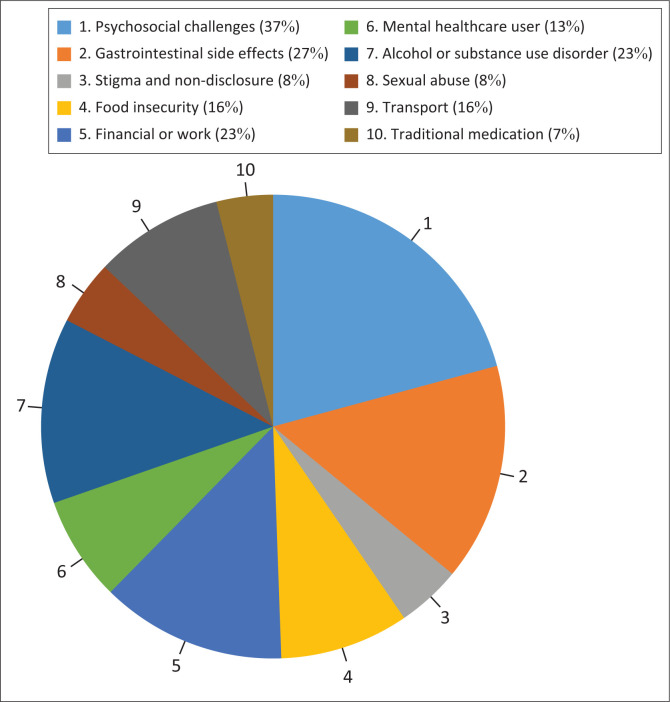
Reasons reported for poor adherence to, or defaulting on, antiretroviral treatment (ART).

In this cohort, there was no correlation between VS and age (*P* = 0.34), gender (*P* = 0.90), living area (*P* = 0.15), employment status (*P* = 0.29), time since diagnosis (*P* = 0.90), duration on ART (*P* = 0.85), previous ART (*P* = 0.90) and current ART regimens (*P* = 0.85). A total of 36 (37.10%) PLHIV had a history of defaulting treatment and 63 PLHIV (64.95%) had a history of poor adherence. The most common reasons reported for poor adherence to, or defaulting on, ART were psychosocial challenges and gastrointestinal side effects (Figure 2).

### Resistance mutations

**FIGURE 3:**
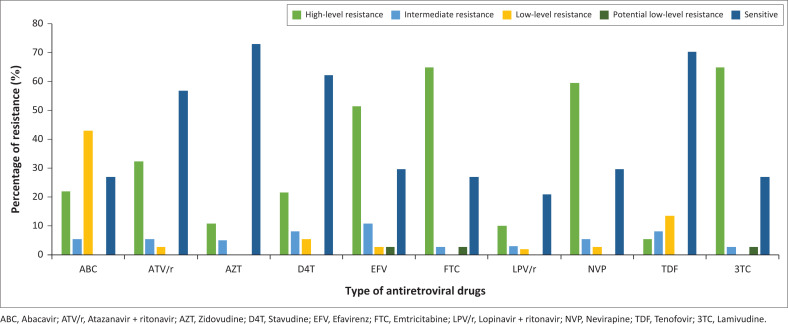
Bar graph showing level of resistance to different antiretroviral treatment (ART) drugs.

Genotypic resistance testing was performed on a total of 37 PLHIV (38.14%). Of these, resistance mutations were found in 31 PLHIV (83.78%), with the most common mutation being M184V of the nucleoside reverse transcriptase inhibitor (NRTI) drug class (64.90%), followed by V82A (32.40%) from the PI major class (Table 3). Other common mutations included *K103N* from the non-nucleoside reverse transcriptase inhibitors (NNRTIs) drug class and the D67N (16.20%) and K70R (16.20%) mutations from the NRTI drug class. Overall, NRTIs and NNRTIs had the most and second-most resistance mutations with 70.30% for NRTIs and 67.60% for NNRTIs. This was followed by resistance mutations to PI minor drugs (16.20%) and PI major drugs (15.20%). Emtricitabine (FTC) and 3TC had the most high-level resistance mutations, whilst AZT and Tenofovir (TDF) were the most sensitive (Figure 3).

**TABLE 3:** Frequencies of resistance mutations found.

PI Major	*n*	%	PI Minor	*n*	%	NRTI	*n*	%	NNRTI	*n*	%
V82A	12	32.4	L10F	7	18.9	M184V	24	64.9	K103N	9	24.3
I54V	11	29.7	K20T	4	10.8	A62V	1	2.7	Y181C	5	13.5
M46I	11	29.7	L24I	4	10.8	D67N	6	16.2	V106M	4	10.8
I50L	2	5.4	L33F	4	10.8	K70R	6	16.2	Y188L	4	10.8
I84V	2	5.4	A71V	3	8.1	T215F	5	13.5	A98G	3	8.1
L76V	2	5.4	L89T	3	8.1	M41L	4	10.8	G190A	3	8.1
I47V	1	2.7	T74S	2	5.4	S68G	4	10.8	H221Y	3	8.1
I50V	1	2.7	F35L	1	2.7	K219E	3	8.1	K103S	3	8.1
N885	1	2.7	G73S	1	2.7	K65R	3	8.1	K101E	3	8.1
-	-	-	K20R	1	2.7	V75M	3	8.1	E138A	2	5.4
-	-	-	K43K	1	2.7	D67G	2	5.4	P225H	2	5.4
-	-	-	L10Y	1	2.7	F77L	2	5.4	V108I	2	5.4
-	-	-	L23F	1	2.7	K219Q	2	5.4	F227L	1	2.7
-	-	-	L23I	1	2.7	T69N	2	5.4	G190S	1	2.7
-	-	-	N83D	1	2.7	V75I	2	5.4	K103K	1	2.7
-	-	-	Q58E	1	2.7	V118I	2	5.4	K101P	1	2.7
-	-	-	T74A	1	2.7	D67D	1	2.7	M230L	1	2.7
-	-	-	T74P	1	2.7	E40D	1	2.7	V179D	1	2.7
-	-	-	V33A	1	2.7	K219R	1	2.7	-	-	-
-	-	-	-	-	-	K66K	1	2.7	-	-	-
-	-	-	-	-	-	K70K	1	2.7	-	-	-
-	-	-	-	-	-	T69DE	1	2.7	-	-	-
-	-	-	-	-	-	T96S	1	2.7	-	-	-
-	-	-	-	-	-	V75F	1	2.7	-	-	-

PI, protease inhibitor; NRTI, nucleoside reverse transcriptase inhibitor; NNRTI, non-nucleoside reverse transcriptase inhibitors.

### Univariate analysis and correlations

Both groups, those with mutations and those without, had similar demographic characteristics. However, the group with mutations had a statistically significant higher median pre- and post-MDT VL compared to those without mutations (*P* < 0.05; Table 4).

**TABLE 4:** Comparison of clinical data between people living with HIV with and without mutations.

Virological characteristics	No mutations					Mutations					*P*
	*n*	Median	IQR	Mean	s.d.	*n*	Median	IQR	Mean	s.d.	
**Pre MDT**											
Pre-MDT VL #1	60	7684	1905–25 550	-	-	25	35 700	3840–92 000	-	-	0.02
Pre-MDT VL #2	62	3850	682–19 600	-	-	28	36 900	8145–129 850	-	-	< 0.001
Pre-MDT VL #3	66	14 350	2830–84 300	-	-	31	41 500	10 600–115 000	-	-	0.06
Pre-MDT CD4	56	-	-	412.05	386.5	25	-	-	301.6	186.82	0.34
**Post MDT**											
Post-MDT VL #1	66	2800	285–18 200	-	-	31	17 300	1300–35 900	-	-	0.02
Post-MDT VL #2	61	592	50–12 600	-	-	29	8560	1099–33 300	-	-	0.02
Post-MDT CD4	24	-	-	385.33	287.39	14	-	-	238.36	143.48	0.11

MDT, multidisciplinary team; VL, viral load; IQR, interquartile range; s.d., standard deviation.

Individuals with neuropsychiatric comorbidities demonstrated a statistically significantly lower rate of mutations at 7.00%, compared to 36.00% in individuals without such comorbidities (*P* = 0.03). However, given the small sample size of the study cohort (where only one individual with mutations had a neuropsychiatric comorbidity), it is important to be cautious when interpreting and generalising these results. There were no significant associations between those who had mutations and comorbidities (excluding neuropsychiatric comorbidities), opportunistic infections, history of defaulting, or any classified reason for defaulting. Third-line treatment was recommended in 17 of the 31 (54.84%) PLHIV with mutations.

## Discussion

The challenges faced by PLHIV are multifactorial and complex. This study showed that the use of an MDT was effective in significantly reducing the VL in PLHIV on 2LART. The value of this intervention is highlighted by the sustainability of VL reduction noted on subsequent measurements. In the post-MDT period, nearly three-quarters of the cohort achieved VS after attending an average of 2.71 MDT sessions. The most common reason for defaulting ART or for poor adherence was the presence of concomitant psychosocial challenges. This supports the work of McLeroy et al., and acknowledges that multiple BPS factors contribute to health outcomes.^45^ Multiple studies confirm that psychosocial challenges directly increase risk-taking behaviour, non-adherence, disease complications, and hospitalisation.^46,47,48^ Additionally, they reduce retention in care and perpetuate HIV transmission.^48,49^ These results emphasise the improved clinical outcomes found with individualised, BPS patient care.

According to the NDoH’s guidelines at the time, 97 PLHIV were eligible for GRT. However, with the use of an MDT, GRT was only performed on 37 PLHIV (38.14%). The remaining 61.86% of the cohort showed a reduction in VL in the post-MDT period, thus no longer required GRT. Based on the currently available literature, VL is seen as a surrogate objective marker for adherence to ART.^50^ Therefore, an educated assumption was made which attributed the improvement in VL to the improvement in adherence to ART.^10,15,16^ Of those who underwent GRT, 31 out of 37 (83.78%) had resistance mutations and only 6 showed no mutations. The most common mutation found, M184V, was consistent with other studies conducted in SA.^51,52,53^ Additionally, NRTIs and NNRTIs were the most common drug classes with resistance mutations in this study and were comparable to a multitude of studies conducted previously in SA.^51,52,54^ Certain mutations, such as M184V, are more likely to be caused by first-line ART exposure and therefore may not be an accurate indication of adherence to 2LART. However, the rationale behind including these data was to allow for comparisons to be made between this cohort and the existing literature in a South African setting. Resistance mutations K70R and D67N, a type two thymidine analogue mutation, accounted for the second-highest number of mutations in the NRTI drug class. These mutations reduce the susceptibility of the virus to AZT.^55^ GRT was avoided in 60 PLHIV who no longer required it due to an adequate reduction in VL post MDT. The average cost of a GRT in SA currently is R5899, therefore R359 280.00 was potentially saved with the use of the MDT intervention. This emphasises the significance of a holistic, BPS approach in patient care to improve patient outcomes. These results suggest that the MDT intervention saved costs by avoiding further unnecessary resistance testing. However, the costs involved in resourcing an MDT intervention, such as the one in this study, would require further analyses to accurately assess the cost-effectiveness of this method of care. Third-line treatment was recommended in 17 of the 31 who had resistance mutations, thus suggesting that the current regimen or a drug change within the current regimen would adequately reduce VL.

The counterintuitive decrease in mean CD4 count post MDT should be viewed in light of the study’s limitations. Pre-MDT CD4 count results were obtained for 81 participants compared to 38 participants from the post-MDT period. This followed clinical guidelines at the time, where CD4 was not routinely tested after achieving initial immune recovery at month 12 post ART initiation. The testing of the CD4 count, after the first annual test at 12 months of ART, was clinician driven, based on clinical suspicion of immunological failure. Thus, the results may be skewed and would require more data to improve accuracy.

Male participants had a higher median VL and lower mean CD4 count compared to female participants in both the pre-MDT and post-MDT periods. This finding is consistent with those of another study conducted in Tshwane, SA, and can be explained by the delayed health-seeking behaviour of men compared to women.^56^ Additionally, women are generally exposed to more opportunistic health intervention drives, such as the Vertical Transmission Prevention programme during pregnancy and in the post-partum period.^57^

Treatment history, including duration of treatment and ART drug class exposure, did not serve as potential predictors for current treatment failure. Surprisingly, employment status was not associated with VS (*P* = 0.29). This finding was not in keeping with those of multiple other studies.^58,59^ There are a multitude of factors that are associated with unemployment which contribute to poor adherence and treatment failure, for example food insecurity and little or no transport funding to attend clinic visits.^60,61^ A larger study, with further in-depth analysis, would be required to explore this association fully to allow for the generalisability of these results.

Those who had a neuropsychiatric comorbidity were less likely to have a resistance mutation on GRT. This finding is not in agreement with other studies and should be interpreted within the study’s limitation of the small sample size. In multiple other studies conducted in high-income countries, concomitant mental health-related disorders were associated with lower adherence rates, higher risk for developing resistance mutations, disease progression, increased hospitalisation, and poorer health outcomes.^62,63,64^ However, Kelly et al. conducted a study in Malawi that found no association between HAND (neuropsychiatric comorbidity) and non-adherence (*P* = 0.69).^65^ Additionally, another study conducted in KZN by Mogambery et al. did not find a significant association between a detectable VL and HAND.^50^ Due to the conflicting evidence, additional and more in-depth studies, with larger sample sizes, may be necessary to explore this association fully.^50,63,64,65^

### Limitations

This study did not have a control group to allow for direct comparison of the results. This makes it difficult to attribute the successful outcomes to the MDT intervention alone. The sample size was significantly smaller due to the nature of the paper-based filing system currently in place at the clinic and all results should be interpreted with this in mind. Every effort was made to collect data of good quality; however, the data collected from existing medical records are subjected to inconsistencies, errors, and missing information. The great success of this MDT is attributed to the resource capacity, training, and expertise of the staff available at NDH. Furthermore, the study was conducted at a district-level healthcare facility, thus it may not be generalisable to other municipalities and districts in SA.

## Conclusion

The use of a targeted, MDT approach in caring for PLHIV failing 2LART resulted in a significant reduction in VL with most participants achieving VS after an average of 2.71 MDT sessions. The MDT identified individuals who would benefit from GRT which resulted in fewer GRTs being done, with high yields for mutations. The resistance mutations found indicated that local resistance patterns reflect those found in other studies conducted in SA. The findings of this study should serve to inform other clinicians, especially those in resource-limited settings, of the benefits of utilising an MDT approach to provide holistic, BPS care for PLHIV to improve clinical outcomes.
